# Recent Advances in PET and Radioligand Therapy for Lung Cancer: FDG and FAP

**DOI:** 10.3390/cancers17152549

**Published:** 2025-08-01

**Authors:** Eun Jeong Lee, Hyun Woo Chung, Young So, In Ae Kim, Hee Joung Kim, Kye Young Lee

**Affiliations:** 1 Department of Nuclear Medicine, Seoul Medical Center, 156 Sinnae-ro, Jungnang-gu, Seoul 02053, Republic of Korea; cateunjeong@naver.com; 2 Department of Nuclear Medicine, Konkuk University Medical Center, Konkuk University School of Medicine, 120-1 Neungdong-ro, Gwangjin-gu, Seoul 05030, Republic of Korea; youngso@kuh.ac.kr; 3 Research Institute of Medical Science, Konkuk University School of Medicine, 120-1 Neungdong-ro, Gwangjin-gu, Seoul 05030, Republic of Korea; 4 Division of Pulmonology, Department of Internal Medicine, Konkuk University Medical Center, Konkuk University School of Medicine, 120-1 Neungdong-ro, Gwangjin-gu, Seoul 05030, Republic of Korea; iakim@kuh.ac.kr (I.A.K.); hjkim@kuh.ac.kr (H.J.K.); kyleemd@kuh.ac.kr (K.Y.L.); 5 Precision Medicine Lung Cancer Center, Konkuk University Medical Center, 120-1 Neungdong-ro, Gwangjin-gu, Seoul 05030, Republic of Korea

**Keywords:** lung cancer, PET, radioligand therapy, FAP, artificial intelligence, radiomics

## Abstract

Lung cancer is one of the most common cancers and the leading cause of cancer-related death worldwide. Despite advancements, the overall survival rate for lung cancer remains between 10% and 20% in most countries. However, recent progress in diagnostic tools and therapeutic strategies has led to meaningful improvements in survival outcomes, highlighting the growing importance of personalized management based on accurate disease assessment. ^18^F-fluorodeoxyglucose positron emission tomography/computed tomography (FDG PET/CT) has become essential in the management of lung cancer, serving as a key imaging modality for initial diagnosis, staging, treatment response assessment, and follow-up evaluation. Recent developments in radiomics and artificial intelligence (AI), including machine learning and deep learning, have revolutionized the analysis of complex imaging data, enhancing the diagnostic and predictive capabilities of FDG PET/CT in lung cancer. However, the limitations of FDG, including its low specificity for malignancy, have driven the development of novel oncologic radiotracers. One such target is fibroblast activation protein (FAP), a type II transmembrane glycoprotein that is overexpressed in activated cancer-associated fibroblasts within the tumor microenvironment of various epithelial cancers. As a result, FAP-targeted radiopharmaceuticals represent a novel theranostic approach, offering the potential to integrate PET imaging with radioligand therapy (RLT). In this review, we provide a comprehensive overview of FDG PET/CT in lung cancer, along with recent advances in AI. Additionally, we discuss FAP-targeted radiopharmaceuticals for PET imaging and their potential application in RLT for the personalized management of lung cancer.

## Simple Summary

Lung cancer is one of the most common cancers and the leading cause of cancer-related death worldwide. FDG PET/CT has become an essential imaging modality in the management of lung cancer. Recent developments in radiomics and artificial intelligence (AI) have revolutionized the evaluation of complex imaging data, enhancing the diagnostic and predictive power of FDG PET/CT in lung cancer. Furthermore, newly introduced fibroblast activation protein (FAP)-targeted radiopharmaceuticals represent a novel theranostic approach, offering the potential to combine PET imaging with radioligand therapy (RLT). In this review, we provide a comprehensive overview of FDG PET/CT in lung cancer, along with recent advances in AI. Additionally, we discuss FAP-targeted radiopharmaceuticals for PET imaging and their potential application in RLT for the personalized management of lung cancer.

## 1. Introduction

Lung cancer is one of the most common cancers and the leading cause of cancer-related death worldwide [1]. Due to differences in the disease pattern and treatment strategies, lung cancer has traditionally been classified into two main types based on cell morphology: non-small cell lung cancer (NSCLC) and small cell lung cancer (SCLC) [2]. Approximately 80% to 85% of lung cancers are NSCLC, which includes three major subtypes—adenocarcinoma (ADC), squamous cell carcinoma (SqCC), and large cell carcinoma. In contrast, SCLC accounts for 10% to 15% of cases and is characterized by its aggressive nature and neuroendocrine features [3]. Despite recent advancements, the overall survival rate for lung cancer remains between 10% and 20% in most countries. However, progress in diagnostic tools and therapeutic strategies has led to meaningful improvements in survival outcomes, emphasizing the growing importance of personalized management based on accurate disease assessment [4].

In the management of lung cancer, ^18^F-fluorodeoxyglucose positron emission tomography/computed tomography (FDG PET/CT) has become a standard imaging modality for initial diagnosis, staging, treatment response assessment, and follow-up evaluation [5]. Simultaneous FDG PET and CT imaging provides complementary functional and anatomical information. Ongoing advancements in imaging technology and methodology have enabled improved visualization, quantification, and interpretation of FDG PET/CT images [6]. Furthermore, recent developments in radiomics and artificial intelligence (AI), including machine learning (ML) and deep learning (DL), have revolutionized the evaluation of complex imaging data. AI can rapidly and precisely analyze large, heterogeneous datasets of varying origin, format, size, and timescale. The integration of AI into FDG PET/CT imaging has the potential to accelerate interpretation and improve diagnostic accuracy and risk stratification in lung cancer [7].

However, FDG is not inherently specific to cancer, as it merely reflects glucose metabolism. Therefore, the development of new cancer-specific radiopharmaceuticals has become necessary [8]. The tumor microenvironment (TME)—formed through interactions between tumor cells and surrounding stromal components via the circulatory system—plays a critical role in tumor development, progression, and treatment response [9]. The TME comprises immune cells, vasculature, extracellular matrix, and cancer-associated fibroblasts (CAFs), with CAFs being among its most prominent components. Fibroblast activation protein (FAP), a type II transmembrane glycoprotein, is highly overexpressed in CAFs across various epithelial cancers, while its expression in normal tissues remains minimal [10]. Although FAP is also involved in inflammatory and fibrotic processes, FAP-targeted radiopharmaceuticals have been proposed as promising alternatives to FDG.

Theranostics integrates PET imaging and radioligand therapy (RLT) to enable personalized patient management. For effective theranostic applications, radioligands must exhibit high target affinity and sufficient tumor retention to maximize tumoricidal effects while minimizing toxicity [11]. FAP-targeted radiopharmaceuticals labeled with ^177^Lu, ^90^Y, or ^225^Ac have demonstrated promising potential for RLT in various malignancies, including lung cancer [12].

In this review, we provide a comprehensive overview of FDG PET/CT in lung cancer, along with recent advances in AI. Additionally, we discuss FAP-targeted radiopharmaceuticals for PET imaging and their potential application in RLT for the personalized management of lung cancer.

## 2. Method—Search Strategy

The PubMed database was searched for articles published between January 2015 and April 2025 using combinations of the following terms: “FDG”, “PET”, “FAP”, “FAPI”, “lung cancer”, “solid tumor”, “incidence”, “mortality”, “detection”, “diagnosis”, “staging”, “response assessment”, “prognosis”, “follow-up”, “radiation therapy”, “immunotherapy”, “radiomics”, “artificial intelligence”, “machine learning”, “deep learning”, “PD-L1”, “gene”, “radioligand therapy”, “^177^Lu”, “^90^Y”, “^225^Ac”, “^211^At”, and “^212^Pb”. All types of articles—including original research, meta-analyses, reviews, editorials, and case reports—were screened.

Both preclinical and clinical studies were reviewed. All clinical studies with prospective or retrospective data were assessed. All authors independently screened the titles and abstracts to evaluate their relevance. Full texts were reviewed when titles and abstracts were deemed relevant. References from the selected articles, including those published outside the specified time window, were also examined. Articles that were irrelevant or not written in English were excluded. Finally, a total of 124 articles were included and are listed in the References section.

**Figure 1:**
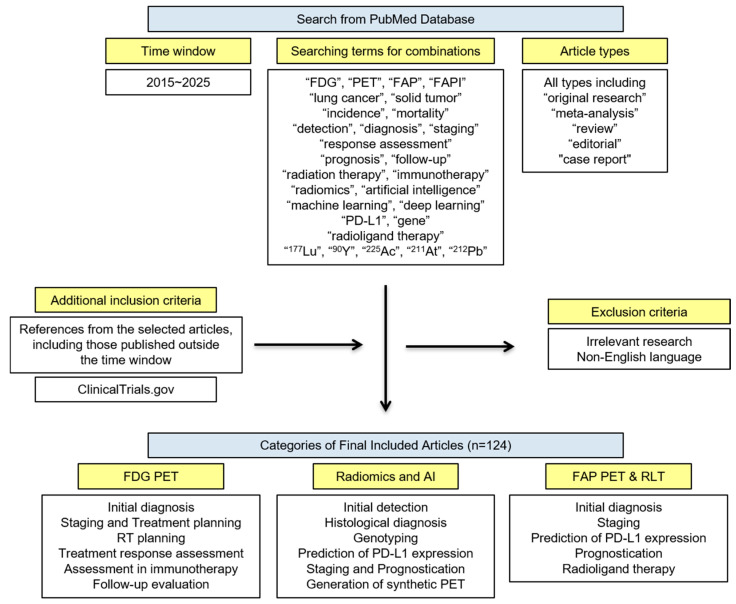
Flow diagram for the search strategy.

ClinicalTrials.gov was searched for active prospective trials using the terms “FAP”, “FAPI”, “lung cancer”, “solid tumor”, “therapy”, “^177^Lu”, “^90^Y”, “^225^Ac”, “^211^At”, and “^212^Pb”. The last search was conducted on 31 May 2025. A flow diagram illustrating the search strategy for this review is presented in Figure 1.

## 3. FDG PET

### 3.1. Initial Diagnosis

Pulmonary lesions measuring less than 3 cm in diameter are classified as nodules, while those larger than 3 cm are categorized as masses [13]. Currently, FDG PET/CT is a well-established imaging modality for evaluating the malignant potential of lung nodules and masses detected by CT [14].

However, FDG uptake is often limited in sub-centimeter or sub-solid nodules, and it may also be reduced in small nodules near the diaphragm due to respiratory motion. In contrast, a solid nodule larger than 8–10 mm with low FDG uptake is more likely to be benign. While FDG PET/CT cannot replace tissue biopsy, it can guide biopsy planning to minimize sampling error [15]. The CT component of FDG PET/CT enhances anatomical localization and provides additional morphological information, increasing diagnostic confidence for malignancy or suggesting alternative benign conditions [16].

In the diagnosis of lung cancer with FDG PET/CT, different cut-off values of the maximum standardized uptake value (SUVmax) have been proposed based on the size of the lung nodule. A prospective multicenter trial including 355 patients with indeterminate solitary pulmonary nodules suggested SUVmax cut-off values of 1.75 (<12 mm, *n* = 133), 2.55 (12–16 mm, *n* = 122), and 3.6 (>16 mm, *n* = 99) [17]. The corresponding areas under the receiver operating characteristic curves (AUCs) with 95% confidence intervals (CIs) for each size group were 0.84 (0.77–0.91), 0.84 (0.77–0.91), and 0.89 (0.82–0.97), respectively. However, variability in SUVmax cut-off values for lung cancer has made the standardization of FDG PET/CT image interpretation challenging, particularly in clinical trials. Furthermore, PET/CT systems utilizing advanced image reconstruction algorithms often produce higher SUVmax values than older-generation systems, even those from the same manufacturer. Consequently, relative uptake analysis—comparing the FDG uptake of a nodule to background activity in the mediastinum or normal lung parenchyma—is recommended to enhance consistency and diagnostic reliability.

According to a meta-analysis of 8511 lung nodules (60% malignant) from 70 studies, FDG PET/CT demonstrated a pooled sensitivity (Sens) of 89% (95% CI, 86–91) and specificity (Spec) of 75% (71–79) for differentiating malignant from benign nodules. However, the diagnostic reliability of FDG PET/CT for lung cancer is reduced in areas endemic for infectious diseases. The adjusted spec in endemic areas was 61% (49–72), which is 16% lower than the 77% (73–80) observed in non-endemic areas. Sens remained unchanged following adjustment for endemic infection status [18]. A study including 514 patients with solitary lung nodules in a tuberculosis-endemic country reported the Sens, Spec, and overall diagnostic accuracy (ODA) of FDG PET/CT for differentiating lung cancer from pulmonary tuberculosis as 96.0%, 48.7%, and 92.0%, respectively [19]. Analyzing the pattern and distribution of FDG uptake with corresponding CT findings is helpful in identifying false-positive (FP) results.

Because FDG uptake tends to increase over time in malignant tumors more than in benign lesions, dual-time-point (DTP) PET imaging—consisting of early and delayed acquisitions—has been suggested to enhance the diagnostic performance of FDG PET/CT relative to standard single-time-point (STP) imaging [20]. According to a meta-analysis of 415 patients from eight studies, DTP imaging yielded a pooled Sens of 79% (95% CI, 74–84) and a spec of 73% (65–79) in the differential diagnosis of pulmonary nodules. In contrast, STP imaging showed a comparable pooled Sens of 77% (72–82) but a lower spec of 59% (51–66) [21]. However, the AUCs were nearly identical (0.824 for DTP vs. 0.822 for STP), raising questions about the additional clinical benefit of DTP imaging. Moreover, the increased complexity and time required for DTP imaging have limited its use in routine clinical practice.

<Summary>

– FDG PET complements CT in differentiating malignant from benign lung nodules.
– FDG uptake is low in sub-centimeter or sub-solid nodules, as well as in small nodules near the diaphragm due to respiratory motion.
– Relative FDG uptake analysis is recommended for the diagnosis of lung cancer.
– In endemic areas for infectious diseases, the diagnostic reliability of FDG PET/CT for lung cancer is reduced.
– DTP PET imaging is not recommended for routine clinical practice.

### 3.2. Staging and Treatment Planning

FDG PET/CT is recommended for staging both NSCLC and SCLC. It provides accurate assessment of nodal and distant metastases by complementing CT scans, guides appropriate treatment, and helps prognosis prediction [22,23,24].

The tumor, node, and metastasis (TNM) staging system is based on the size and extent of the primary tumor (T), involvement of regional lymph nodes (N), and presence of distant metastases (M). Since the 8th edition in 2017, the application of this staging system has been extended to SCLC and bronchopulmonary carcinoid tumors, as well as NSCLC [25]. Despite the adoption of the TNM system for SCLC, it is still commonly categorized into two clinical stages—limited stage (LS) and extensive stage (ES)—according to the feasibility of radiation therapy (RT) [26].

**Figure 2:**
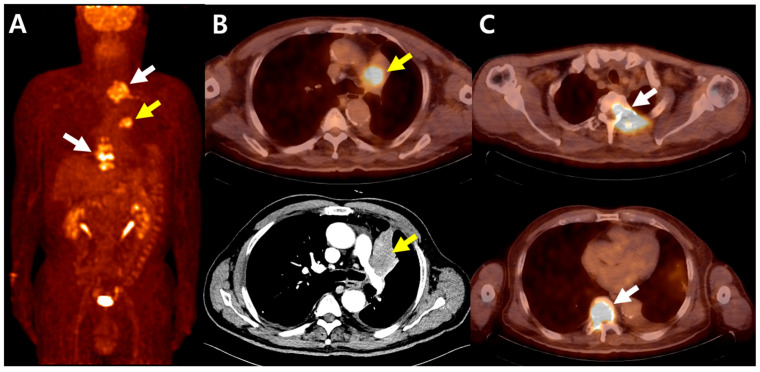
FDG PET/CT and contrast-enhanced CT images of a 67-year-old man with lung adenocarcinoma. (**A**) Maximum-intensity projection (MIP) images demonstrate intense FDG uptake in the left upper lobe lung mass (yellow arrow) and in spinal lesions at T2–T4 and T8–T11 (white arrows). (**B**) Axial FDG PET/CT and contrast-enhanced CT images show a central lung mass in the left upper lobe with distal atelectasis. FDG PET/CT reveals a lack of FDG uptake in the atelectatic lung, while contrast-enhanced CT shows no clear demarcation between the mass and distal atelectasis. (**C**) Axial FDG PET/CT images demonstrate increased FDG uptake in the T2–T4 and T8–T11 vertebral lesions.

For T staging of lung cancer, CT is generally more reliable than FDG PET/CT, as intense FDG uptake may overestimate invasion to adjacent organs due to spillover effects [27]. However, FDG PET/CT is useful for distinguishing lung cancer from rounded or post-obstructive atelectasis, as FDG uptake in atelectasis is typically lower than that in tumors but higher than in normal lung parenchyma [28]. A representative case of FDG PET/CT for staging lung cancer is shown in Figure 2.

Accurate N staging is essential for assessing operability in lung cancer without distant metastases. A systematic review involving 7368 patients with NSCLC reported that the Sens and spec for detecting mediastinal nodal metastasis of chest CT were 55% and 81%, respectively. For FDG PET/CT (or PET), Sens and spec improved to 77% and 86%, respectively [29]. In addition, a meta-analysis evaluated the diagnostic performance of FDG PET/CT for N staging in NSCLC based on the method of FDG uptake analysis. When nodal uptake was compared to background mediastinal activity, the Sens and spec of FDG PET/CT for detecting mediastinal nodal metastasis were 77.4% and 90.1%, respectively, However, when a threshold of SUVmax 2.5 was applied, Sens and spec were 81.3% and 79.4%, respectively [30]. While SUVmax is important for image interpretation, the avidity of the primary tumor and other clinical factors should also be considered [31].

FDG PET/CT has shown a false-negative (FN) rate of 10–21% and a FP rate of 45–48% for N staging [32,33]. FN nodal FDG uptake has been reported in some large tumors with high FDG uptake [34]. FP nodal FDG uptake commonly occurs in the presence of infectious lung diseases or centrally located tumors [35]. These FP findings reduce the spec of FDG PET/CT by mimicking nodal metastases. Sens is also affected, as inflammatory nodes may conceal metastatic involvement. Integrating structural and metabolic information from FDG PET/CT can help minimize both FN and FP interpretations [36].

Endobronchial ultrasound-guided fine needle aspiration (EBUS-FNA) has recently become the standard method for preoperative N staging in NSCLC [37]. The combination of EBUS-FNA and FDG PET/CT improved both spec and positive predictive value (PPV) to 100%, with an ODA of 97%, compared to 90% spec, 87% PPV, and 88% ODA with FDG PET/CT alone. The Sens remained unchanged at 95% with the combination [38]. Peeters et al. calculated that the addition of EBUS-FNA could reduce the FN rate of FDG PET/CT from 13% to 3% in enlarged lymph nodes, and from 6% to 1% in normal-sized nodes. However, in the PET-positive but EBUS-negative (PET+/EBUS−) nodes, the FN rate of EBUS remained 14–16%. Thus, when PET findings strongly indicate mediastinal involvement, PET+/EBUS− nodes should be considered in the treatment planning [39].

For M staging of NSCLC, a meta-analysis of 360 patients from four studies reported pooled Sens and spec of FDG PET/CT as 0.77 (95% CI, 0.47–0.93) and 0.95 (0.92–0.97), respectively. Although the Sens was relatively low, the AUC was 0.96 (0.94–0.97), indicating excellent overall diagnostic performance [40].

FDG PET/CT demonstrated a Sens of 0.92 (95% CI, 0.88–0.95) and spec of 0.98 (0.97–0.98) for the detection of bone metastases. This diagnostic performance exceeds that of bone scintigraphy, particularly when PET and CT findings are concordant, yielding a PPV of 98% [41]. In the evaluation of adrenal metastases, potential FPs (e.g., adrenal hyperplasia, functioning adenoma, and tuberculosis) and FNs (e.g., very small lesions, hemorrhagic or necrotic metastases) should be considered [42]. FDG PET/CT is useful in guiding pathological confirmation by identifying viable metastases with high FDG uptake [43]. However, MRI remains the primary imaging modality for detecting brain metastases, due to the high physiological FDG uptake in normal brain tissue.

By optimizing treatment with accurate staging, FDG PET/CT may reduce healthcare costs in patients with lung cancer. Management strategies have been reported to change in 13% to 26% of NSCLC cases following its use [44,45]. FDG PET/CT can upstage NSCLC by detecting metastases that are occult on CT or downstage suspected CT lesions by demonstrating low FDG uptake [46]. In a study by Fischer et al., 98 NSCLC patients were randomly assigned to preoperative staging with either FDG PET/CT or conventional methods. Thoracotomy was performed in 60 patients in the FDG PET/CT group and 73 in the conventional staging group (*p* = 0.004). Among these, 21 thoracotomies in the PET/CT group and 38 in the conventional group were considered futile (*p* = 0.05) [47]. The cost-effectiveness of FDG PET/CT in the management of NSCLC is largely attributed to reducing futile thoracotomies [48].

For SCLC evaluation, FDG PET/CT with brain MRI is recommended when CT suggests LS disease, whereas further staging with FDG PET/CT is optional for suspected ES disease [49]. A meta-analysis of 369 patients with SCLC from 12 studies, FDG PET/CT (or PET) demonstrated pooled Sens and spec of 97.5% (95% CI, 94.2–99.2) and 98.2% (94.9–99.6), respectively, for the detection of ES disease [50]. Another meta-analysis of 277 SCLC patients from 6 studies found that FDG PET/CT changed binary staging—from LS to ES or vice versa—in 15% (95% CI, 9–21) of cases, when compared to conventional imaging methods. Nonetheless, its utility in detecting brain metastases in SCLC remains limited [51]. Because of the potential for FP results, treatment decisions based on FDG PET/CT findings should be confirmed pathologically. When FDG PET/CT is not available, bone scintigraphy is recommended to assess for LS disease [22].

<Summary>

– FDG PET/CT is recommended for staging both NSCLC and SCLC, particularly for N and M staging.
– Combining EBUS-FNA with FDG PET/CT significantly improves the specificity of N staging.
– The cost-effectiveness of FDG PET/CT in NSCLC largely depends on reducing futile thoracotomies.
– FDG PET/CT significantly affects binary staging decisions in SCLC.

### 3.3. RT Planning

With the accurate delineation of disease extent provided by FDG PET/CT, advanced RT techniques can deliver successful definitive treatment for both NSCLC and SCLC [52]. FDG uptake contributes to identifying tumor-bearing tissues, while CT defines tumor margins. A meta-analysis reported that incorporating FDG PET/CT changed target volume definition in 36% (95% CI, 16–62) of all patients—43% (35–51) in NSCLC and 26% (14–44) in SCLC. Moreover, it led to a change in treatment intent in 20% (9–39) of patients—22% (18–26) in NSCLC and 9% (4–18) in SCLC [53].

In a randomized controlled trial involving patients with locally advanced, inoperable NSCLC, RT planning based on strictly FDG PET-guided small target volumes was compared with that based on conventional criteria-guided large target volumes [54]. The outcome of the FDG PET-guided target group was non-inferior to that of the conventional criteria-guided target group. Among patients who received chemoradiotherapy, the cumulative incidence for locoregional progression was 14% (95% CI, 5–21) at 1 year, 20% (10–29) at 2 years, and 23% (12–32) at 3 years in the FDG PET-guided target group and 29% (17–38) at 1 year, 39% (27–50) at 2 years, and 42% (30–53) at 3 years in the conventional target group. Hazard ratio (HR) was 0.57 (95% CI, 0.30–1.06), confirming non-inferiority within the predefined margin of 1.25. Overall survival (OS) and progression-free survival (PFS) were similar between the two groups. Median OS was 2.41 (95% CI, 1.80–3.26) years in the FDG PET-guided target group and 3.08 (1.80–3.61) years in the conventional target group. Median PFS was 0.92 (0.73–1.40) years in the FDG PET-guided target group and 0.85 (0.73–1.02) years in the conventional target group. Acute and late treatment-related toxicities were also comparable between the groups.

In patients with LS SCLC, OS and PFS did not significantly differ between those staged with additional FDG PET/CT and those staged with conventional imaging (HR = 0.87 [95% CI, 0.70–1.08] for OS, *p* > 0.05; HR = 0.87 [0.71–1.07] for PFS, *p* > 0.05) [55]. Patients staged with additional FDG PET/CT received lower radiation doses to normal tissues—including the lung, heart, and esophagus—compared to those staged by conventional imaging. The incidences of acute and late RT-related toxicities did not differ significantly between the two groups.

<Summary>

– FDG PET-guided RT can deliver successful definitive treatment for both NSCLC and SCLC.

### 3.4. Treatment Response Assessment

The Response Evaluation Criteria in Solid Tumors (RECIST), which assess tumor size before and after treatment, have been widely accepted for evaluating treatment response in oncology clinical trials and routine practice since the development in 2000 and the release of the updated version 1.1 in 2009 [56]. Treatment response is categorized as complete response (CR: disappearance of all target lesions), partial response (PR: ≥30% decrease in the sum of the longest diameters of target lesions from baseline), progressive disease (PD: ≥20% increase in the sum of the longest diameters of target lesions or the appearance of new malignant lesions), and stable disease (SD: neither sufficient shrinkage for PR nor sufficient increase for PD). In RECIST 1.1, FDG PET/CT is recommended as a complementary modality to CT or MRI for assessing new lesions during follow-up to determine PD [57].

However, size measurements of lung cancer based solely on CT can lead to intra- and inter-observer variability and misinterpretation of tumor response. This is particularly true when lesions have irregular or spiculate shapes, are associated with anatomical changes such as atelectasis, inflammation, or post-radiation fibrosis, or when tumors undergo central necrosis [58]. Because changes in cellular metabolism occur more rapidly than changes in tumor size, evaluating treatment response based on metabolic activity has been proposed [59]. Furthermore, 41% discordance was observed between CT-based RECIST and histopathologic responses in NSCLC patients after neoadjuvant chemotherapy [60].

The PET Response Criteria in Solid Tumors (PERCIST) were introduced in 2009 to assess metabolic tumor response both qualitatively and quantitatively [61]. PERCIST evaluates metabolic activity by measuring the peak SUV normalized for lean body mass (SUL) within a 1 mL spherical volume of interest (VOI) in the tumor with the highest FDG uptake. The PERCIST categories for treatment response are as follows: (1) complete metabolic response (CMR: complete resolution of FDG uptake), (2) partial metabolic response (PMR: a decrease of ≥30%, with a minimum absolute drop of 0.8 in peak SUL between the most intense evaluable lesions at baseline and follow-up), (3) stable metabolic disease (SMD: a change in peak SUL of <30% in either direction), and (4) progressive metabolic disease (PMD: an increase of ≥30%, with a minimum absolute rise of 0.8 in peak SUL between target lesions, or the appearance of new lesions). To assess CMR, background normal activity is measured as the mean SUL in the liver within a 3-cm-diameter spherical VOI or in the descending thoracic aorta blood pool within a cylindrical VOI measuring 1 cm in diameter and 2 cm in length. To reduce variability caused by patient body weight, image noise, and filtering, SUL is used instead of single-pixel SUVmax, which is the most commonly used PET imaging metric in routine clinical practice [62].

In a prospective study of 35 patients with advanced NSCLC treated with chemotherapy, PERCIST 1.0 was found to be more sensitive and accurate than RECIST 1.1 in evaluating therapeutic response [63]. However, PERCIST 1.0 has not been widely adopted in routine clinical practice due to challenges such as the lack of a clear definition for unequivocal progression of non-target lesions, unclear criteria for categorizing new lesions, and the additional effort required to obtain SUL measurements.

Because post-treatment inflammation often leads to FP FDG uptake, the timing of FDG PET/CT after treatment is crucial for accurate response assessment. During chemotherapy, although the most significant response is typically observed 3–4 weeks after initiation, an interval of 1–2 weeks may be sufficient for evaluation. Postoperative inflammatory changes can persist for up to 6 months following surgical resection. Areas treated with stereotactic body RT (SBRT) may exhibit well-defined, intense FDG uptake for as long as 6 months. With recent advances in immunotherapy, a wide range of immune-related adverse events has been reported in association with immunotherapeutic agents. Recommended time intervals for treatment response assessment using FDG PET/CT are summarized in Table 1 [55,58,64,65].

<Summary>

– PERCIST may provide a more accurate assessment of treatment response than RECIST. However, its clinical adoption remains limited due to several challenges.
– Accurate assessment of treatment response depends on the appropriate timing of FDG PET/CT after treatment.

### 3.5. Response Assessment in Immunotherapy

During immunotherapy, atypical treatment responses have been reported more frequently than with conventional therapies. These atypical responses can be classified into four categories: pseudoprogression, hyperprogression, durable response, and dissociated response [66].

Pseudoprogression refers to a transient increase in tumor size or the appearance of new lesions due to immune cell infiltration or a delayed immune response. Hyperprogression is characterized by rapid and severe disease progression. Durable response indicates sustained tumor control even after discontinuation of immunotherapy. Dissociated responses describe a pattern in which some lesions progress while others regress, resembling the mixed response observed with chemotherapy. As a result, modified criteria such as immune RECIST (iRECIST) and immune-modified RECIST (imRECIST) have been proposed, which recommend extended observation—typically at least 4 weeks—to confirm true PD [67,68].

The use of FDG PET/CT in response assessment after immunotherapy has also been subject to challenges. Humbert et al. prospectively evaluated 50 patients with metastatic NSCLC who were treated with pembrolizumab or nivolumab and underwent FDG PET/CT for treatment response assessment. Among them, 19 patients showed PMD at 7 weeks post-treatment but did not exhibit clinical deterioration and continued immunotherapy. A follow-up FDG PET/CT scan at 3 months revealed that 5 of these patients had immune dissociated responses and 6 had pseudoprogression, both of which were considered to reflect clinical benefit from immunotherapy [69]. Therefore, when differentiation between true progression and pseudoprogression is uncertain, a follow-up FDG PET/CT is recommended. This approach aligns with current consensus, as no validated imaging method can reliably differentiate the two based on a single scan. Accordingly, in clinically stable patients, treatment should be continued, as they may ultimately derive clinical benefit and achieve a favorable response at a later time-point [70].

**Figure 3:**
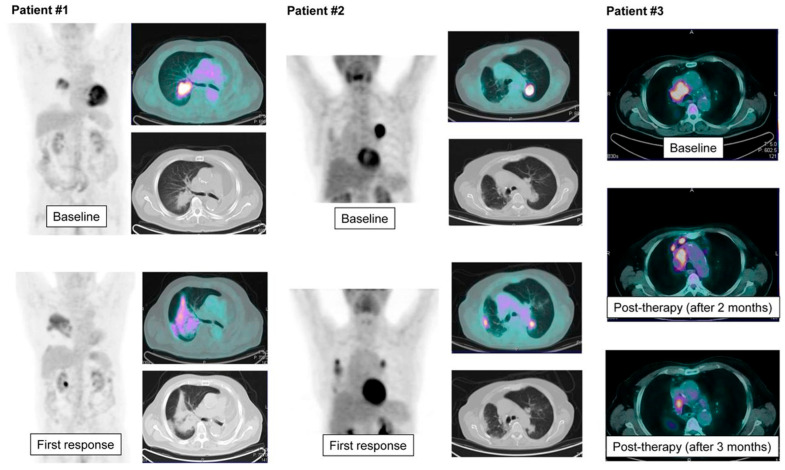
Representative FDG PET/CT images of 3 patients showing discordant response at CT-based and FDG PET/CT-based evaluation or between PERCIST and imPERCIST. Patient 1 was classified as PD with RECIST because of significant increase in dimensions of perihilar lesion in right lung; however, marked reduction in peak SUL allowed us to classify this patient as PMR according to both PERCIST and imPERCIST. In contrast, patient 2 showed marked lesion shrinkage, but lesion metabolism was substantially stable (possibly from inflammatory infiltration) or even mildly increased in the case of right pleural metastasis. This patient was classified as SMD with both PERCIST and imPERCIST, but the response was more evident on CT images. Patient 3 was differently classified by PERCIST and imPERCIST. At first response (2 mo after therapy), he was classified as SMD by imPERCIST; however, because of new lesions, was considered PERCIST PMD. After 1 mo more, new lesions disappeared and lesions present since baseline showed a significant reduction in both dimensions and metabolism. (Reprinted/adapted with permission from Ref. [71]. Copyright © Society of Nuclear Medicine and Molecular Imaging).

Rossi et al. compared CT-based criteria and FDG PET/CT-based criteria for initial response evaluation to nivolumab in 72 patients with advanced NSCLC [71]. The agreement between the two methods was low. For OS, CT-based criteria were more reliable in distinguishing responders from nonresponders. However, a subset of patients classified as having a PMR had longer OS than those classified as having a PR. Representative images of patients with discordant responses between evaluation criteria are shown in Figure 3 [71].

<Summary>

– Follow-up FDG PET/CT is recommended during immunotherapy to distinguish atypical treatment responses.

### 3.6. Follow-Up Evaluation

According to current clinical guidelines for lung cancer, routine follow-up imaging to detect recurrence or a second primary cancer after curative surgery or RT should be performed using CT [72,73]. The use of FDG PET/CT is recommended when malignancy is suspected based on clinical or radiologic findings.

FDG PET/CT has shown equivalent or superior diagnostic performance to clinical examination and chest CT for detecting lung cancer recurrence—either locoregional or distant—with reported Sens of 97–100% and spec of 62–100% [65]. A meta-analysis of 1035 patients from 13 studies evaluated the pooled Sens and spec of FDG PET/CT and conventional imaging (including chest radiography, CT, bone scintigraphy, and MRI) for detecting recurrent lung cancer [74]. The Sens and spec of FDG PET/CT were 0.90 (95% CI, 0.84–0.95) and 0.90 (0.87–0.93), respectively, compared to 0.78 (0.71–0.84) and 0.80 (0.75–0.84) for conventional imaging. FDG PET/CT demonstrated significantly better performance in both Sens and spec (all *p* < 0.05), with higher AUC (0.953 vs. 0.848, respectively).

Antoniou et al. reported that FDG PET/CT detected recurrence after completion of primary treatment in 43.7% (107/245) of patients without prior clinical suspicion, and ruled out recurrence in 15.2% (37/243) of patients with prior clinical suspicion [75]. Among patients with clinical suspicion of recurrence, the median OS was 38.6 months in those with positive PET findings and 74.6 months in those with negative PET findings (*p* = 0.011). In the routine surveillance group, the median OS was 28.3 months for patients with positive PET and 93.7 months for those with negative PET (*p* < 0.001).

Marcus et al. also demonstrated the clinical value of serial FDG PET/CT in patients with lung cancer following primary treatment [76]. At least four follow-up FDG PET/CT scans were performed in 85 patients, of whom 47 (55.3%) died during the study period. Local or distant recurrence was detected in 44.3% (97/219) without prior clinical suspicion, while recurrence was ruled out in 24.2% (16/66) with prior suspicion. FDG PET/CT resulted in a change in treatment in 28.1% (80/285)—initiating treatment in 20.4% (58/285), modifying it in 5.6% (16/285), and discontinuing it in 2.1% (6/285).

<Summary>

– FDG PET/CT has demonstrated diagnostic value for routine follow-up imaging in lung cancer.

## 4. Radiomics and AI

The suffix “-omics” is commonly used in biomedical fields to represent the extraction of valuable information from large datasets. Radiomics refers specifically to the extraction of quantitative data from medical imaging [77]. Traditionally, medical images have been interpreted descriptively and qualitatively, with the exception of size measurements. However, radiomics allows the extraction of high-dimensional quantitative features—such as texture heterogeneity, shape descriptors, and intensity patterns—using advanced mathematical analysis. This approach is based on the assumption that digital medical images contain disease-specific information that may not be recognizable to the human eye but can be identified through sophisticated computational techniques [78]. After feature extraction, the value of radiomic data can be further enhanced through integration with AI models for various clinical applications. Consequently, FDG PET/CT radiomic features (RFs) combined with AI models have been extensively investigated in lung cancer management [79].

### 4.1. Initial Detection

The detection of small lung nodules using FDG PET/CT remains difficult due to limited spatial resolution. Recent advancements in DL have led to the development of effective algorithms for lung nodule detection, particularly those based on convolutional neural network (CNN) techniques such as U-Net and its variants [80].

Protonotarios et al. reported integrating a few-shot learning framework—designed to learn new objects from a small number of examples, similar to human learning—into the U-Net architecture. This approach achieved a 39% reduction in error rate for lung cancer segmentation on PET/CT imaging compared to the original U-Net [81]. Park et al. proposed a two-stage U-Net architecture (global + regional U-Nets) to segment lung cancers with low FDG uptake, contrasting with the conventional one-stage 3D U-Net (global U-Net only). The two-stage U-Net outperformed the one-stage U-Net, achieving a mean Dice coefficient of 0.78 vs. 0.74 (*p* < 0.05), without a significant difference in computation time (less than 1.5 s) [82].

DL–based PET reconstruction techniques—ranging from image denoising and direct sinogram-to-image reconstruction to hybrid models integrating neural networks with traditional iterative methods—have enhanced the accuracy of lung nodule detection [83]. A modified encoder–decoder U-Net architecture has been effectively applied for denoising and partial volume correction of PET images [84]. Furthermore, automated detection performance for small pulmonary nodules was significantly improved using block-sequential regularized expectation maximization (AUC 0.848, 95% CI 0.828–0.869) compared to conventional ordered-subsets expectation maximization (AUC 0.796, 0.772–0.869; *p* = 0.001) [85].

DL models were developed for fully automated whole-body tumor segmentation on PET/CT. Manual segmentation is time-consuming, labor-intensive, and reliant on expert input—especially in patients with a high tumor burden. A DL model based on the nnU-Net architecture achieved a median true positive rate of 0.75, a median PPV of 0.92, a median Dice similarity coefficient of 0.81, and a median false discovery rate of 0.08 in patients with lung cancer [86]. Constantino et al. reported that a 3D U-Net model incorporating additional multi-angle MIP PET images, alongside standard PET/CT, reduced the false discovery rate in fully automated lesion segmentation—0% vs. 12% in the internal test set and 0% vs. 15% in the external test set—compared to a model using standard PET/CT alone [87].

<Summary>

– AI approaches have been applied to PET/CT for lung nodule detection and whole-body tumor segmentation.

### 4.2. Histological Diagnosis

Noninvasive approaches to differentiating tumor pathology reduce the need for invasive biopsies and enable more precise guidance of targeted therapies. An ML-based automated analysis method was developed for diagnosing early-stage lung cancer using RFs from FDG PET/CT [88]. The model achieved accuracies of 82.0% in the testing group and 82.1% in the validation group. The FDG PET/CT–based model had an AUC of 0.89 (95% CI, 0.86–0.91), compared to 0.83 (0.80–0.87) for the CT-based model, highlighting the potential of FDG PET–derived RFs in distinguishing benign from malignant lung nodules. Lai et al. reported that a 3D high-resolution representation DL model (HRNet) significantly outperformed a conventional residual network (ResNet) model for the automated differentiation of lung nodules on FDG PET/CT, with AUCs of 0.781 (95% CI, 0.755–0.834) vs. 0.652 (0.582–0.737), respectively (*p* = 0.004) [89]. Furthermore, the FDG PET/CT–based HRNet model achieved a higher AUC than the CT-only HRNet model (0.781 vs. 0.725), although the difference was not statistically significant (*p* = 0.742).

Predicting histological subtypes of NSCLC is essential for treatment planning. Shen et al. analyzed four tumor subregions—rather than the entire tumor—to better capture intratumoral heterogeneity [90]. RFs extracted from FDG PET/CT subregions, combined with a support vector machine (SVM) classifier using a radial basis function kernel, differentiated ADC from SqCC with a sensitivity of 0.854, specificity of 0.876, ODA of 0.862, and an AUC of 0.916. Zhao et al. reported the effectiveness of FDG PET/CT–based DL models for differentiating NSCLC subtypes (ADC vs. SqCC) [91]. Among the models, the MobileNet v2 model, trained on 1280 extracted deep learning radiomic features, achieved the best performance with AUCs of 0.997 in the training set, 0.744 (95% CI, 0.589–0.900) in the internal test group, and 0.767 (95% CI, 0.617–0.917) in the external test group.

<Summary>

– FDG PET/CT-based models can differentiate benign from malignant lung nodules, as well as ADC from SqCC.

### 4.3. Genotyping

Lung cancer patients with mutations in specific driver genes—such as epidermal growth factor receptor (EGFR), Kirsten rat sarcoma viral oncogene homolog (KRAS), or tumor protein p53 (TP53)—are potential candidates for molecular targeted therapies using matched tyrosine kinase inhibitors [92]. Yang et al. demonstrated ML classifiers combining clinical data, FDG PET/CT metabolic parameters, and RFs to differentiate EGFR wild type, exon 19, and exon 21 mutations in NSCLC [93]. The SVM model outperformed decision tree (DT) and random forest models. In the training cohort, SVM achieved AUCs of 0.881, 0.849, and 0.851; in the validation cohort, 0.926, 0.859, and 0.805, respectively. DT and random forest models showed lower AUCs across all mutation subtypes and cohorts. For the DT model, AUCs were 0.855, 0.879, and 0.780 in the training cohort, and 0.887, 0.822, and 0.776 in the validation cohort. The random forest model showed AUCs of 0.829, 0.783, and 0.826 in the training cohort, and 0.811, 0.728, and 0.713 in the validation cohort

Hinzpeter et al. developed an FDG PET/CT-based radiogenomics model to detect TP53, KRAS, and EGFR gene mutations in NSCLC [94]. The optimal combination of RFs maximizing the Youden Index (Sensitivity + Specificity − 1) was selected using an all-possible-subsets logistic regression approach for each imaging modality. The highest Youden Index was 0.70 (95% CI, 0.50–0.89) for TP53 using combined FDG PET/CT RFs, 0.57 (0.43–0.83) for KRAS using FDG PET RFs only, and 0.60 (0.44–0.97) for EGFR using CT RFs only.

<Summary>

– FDG PET/CT-based models can assist in identifying driver gene mutations in NSCLC.

### 4.4. Prediction of PD-L1 Expression

Immunotherapy targeting the programmed death-1/programmed death-ligand 1 (PD-1/PD-L1) axis has transformed the management of various malignancies, particularly NSCLC. Currently, PD-L1 expression assessed by immunohistochemistry (IHC) is the only Food and Drug Administration-approved biomarker. However, due to tumor heterogeneity, IHC alone is not sufficient for accurately predicting therapeutic response. Thus, a comprehensive multiparametric approach is required to better identify patients most likely to benefit from immunotherapy [95].

An ML classification model based on FDG PET/CT RFs was developed to predict high PD-L1 expression (≥50% of tumor cells by IHC staining) in patients with NSCLC [96]. Three selected features—FBS Szm_sze, HHH Loc_peak_local, and HHH Cm_Corr—were incorporated into a multivariate model to determine the optimal combination for identifying strong PD-L1 expressors. The best performance was observed with linear discriminant analysis and linear SVM models, achieving an AUC of 0.82 ± 0.02, Sens of 81 ± 17%, and spec of 82 ± 6%.

A DL model integrating FDG PET/CT images and clinical data was developed to predict PD-L1 expression status in patients with advanced NSCLC [97]. A small residual convolutional network was employed for the noninvasive assessment of PD-L1 expression. The model significantly distinguished between PD-L1–positive and –negative patients, achieving AUCs ≥ 0.82 across training, validation, and two external test cohorts. Additionally, the model identified patients likely to experience durable clinical benefit (PFS > 6 months) from immune checkpoint inhibitor therapy, with AUCs of 0.70 (95% CI, 0.63–0.77; *p* < 0.001) and 0.72 (0.62–0.84; *p* = 0.014) in retrospective and prospective cohorts, respectively.

Da-Ano et al. investigated the automatic prediction of PD-L1 status in NSCLC patients using DL models [98]. Three DL architectures—ResNet, DenseNet, and EfficientNet—were evaluated in combination with different input strategies: CT only, FDG PET only, early fusion of FDG PET and CT within the network, and late fusion of the two modalities. Models using early fusion of FDG PET and CT images consistently outperformed other approaches, achieving AUCs ≥ 0.79 for PD-L1 prediction. Among the architectures, ResNet and DenseNet generally outperformed EfficientNet.

<Summary>

– FDG PET/CT–based models can help predict PD-L1 expression in NSCLC.

### 4.5. Staging and Prognostication

An FDG PET/CT-based radiomics ML model was developed to predict visceral pleural invasion, which is a key factor in distinguishing T1 from T2 tumors in T staging, in patients with clinical stage IA NSCLC [99]. RFs were extracted from both intratumoral and peritumoral regions. The model achieved AUCs of 0.879 (95% CI, 0.838–0.920), 0.846 (0.748–0.943), and 0.745 (0.606–0.884) in the training, internal validation, and external validation cohorts, respectively.

Occult nodal metastasis in patients with clinical N0 stage NSCLC was predicted using an FDG PET/CT-based cross-modal DL model [100]. For predicting occult N1 disease, the model achieved AUCs of 0.958 (95% CI, 0.923–0.992) in the validation set, 0.879 (0.813–0.946) in the external cohort, and 0.914 (0.877–0.949) in the prospective cohort. For occult N2 disease, AUCs were 0.942 (0.911–0.973), 0.875 (0.820–0.930), and 0.919 (0.886–0.942), respectively. The cross-modal DL model significantly outperformed single-modal PET and CT DL models, a clinical model, and assessments by both senior and junior physicians.

An FDG PET/CT–based DL grading model was developed to predict recurrence risk in patients with clinical stage I invasive lung adenocarcinoma [101]. The model achieved AUCs of 0.862 (95% CI, 0.830–0.894), 0.844 (0.803–0.885), and 0.851 (0.819–0.882) in the validation set, external cohort, and prospective cohort, respectively. The DL grading model significantly outperformed FDG PET, CT, and clinical models (DeLong’s test; all *p* < 0.05). High-risk patients, as identified by the model, showed significantly worse prognosis compared to low-risk patients (3-year OS: 92.1% vs. 98.0%; 3-year recurrence-free survival [RFS]: 76.2% vs. 92.8%; both *p* < 0.001). Among high-risk patients, those who underwent lobectomy had better outcomes than those who received sublobar resection (3-year OS: 93.5% vs. 87.8%, *p* = 0.085; 3-year RFS: 78.4% vs. 69.2%, *p* = 0.038). In addition, systemic nodal dissection was associated with superior prognosis compared to limited nodal dissection (3-year OS: 94.8% vs. 84.4%, *p* = 0.001; 3-year RFS: 78.5% vs. 69.9%, *p* = 0.041).

For predicting local or distant recurrence after SBRT in patients with early-stage NSCLC, an FDG PET/CT-based ML radiomics model outperformed models based on clinical parameters [102]. The AUC values for the clinical, radiomic, and combined (clinical + radiomic) models were 0.58 (95% CI, 0.52–0.64), 0.74 (0.69–0.80), and 0.67 (0.61–0.73), respectively, in the training set, 0.53 (0.46–0.60), 0.79 (0.72–0.84), and 0.67 (0.60–0.73), respectively, in the internal testing set, and 0.51 (0.38–0.64), 0.83 (0.71–0.91), and 0.56 (0.43–0.68), respectively, in the external testing set. Only the radiomics model significantly stratified patients into high- and low-risk groups for local or distant recurrence in both the internal and external testing sets.

<Summary>

– FDG PET/CT–based models can support staging and prognostication in NSCLC.

### 4.6. Generation of Synthetic PET Image

Although PET provides valuable clinical information, it remains costly and less accessible compared to CT. Generating meaningful medical images is more challenging than generating natural images, as clinical diagnosis often depends on detecting subtle changes in the appearance of complex anatomical structures. Recently, the emergence of generative DL models has enabled cross-modality imaging synthesis, making it possible to generate PET images from CT data [103].

**Figure 4:**
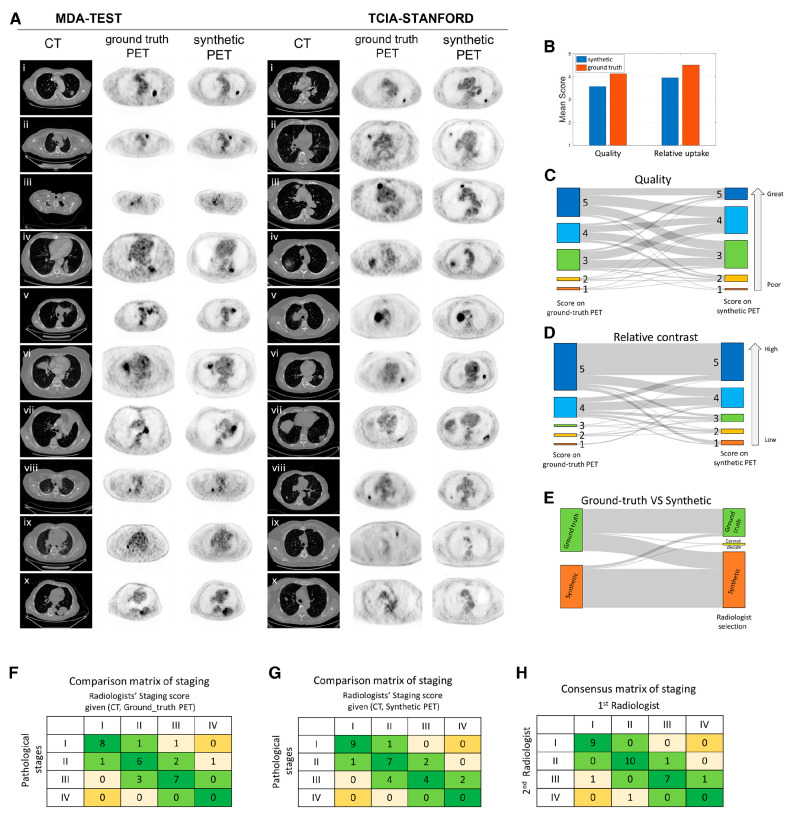
Validation of imaging signal fidelity and cancer staging performance by radiologists. (**A**) Presentation of synthetic PET images with ground-truth PET in MD Anderson (MDA)-TEST and The Cancer Imaging Archive (TCIA)-STANFORD testing cohorts. The first three columns from left to right for each cohort correspond to CT, ground-truth, and synthetic PET images. The PET images are shown inversely with the normalized window of SUV in [0, 3]. Therefore, the completely black color in tumors indicates that the tumor had uptake with maximum SUV value of at least 3. (**B**) The radiologists’ score on quality and relative uptake of lung region in task 1 of the imaging quality test. (**C**) Alluvial plot shows the radiologists’ scoring on imaging quality difference using paired PET scans. (**D**) Alluvial plot shows the radiologists’ scoring on tumor contrast difference using paired PET scans. (**E**) Alluvial plot shows the radiologists’ reading of ground-truth vs. synthetic using paired PET scans. (**F**) Comparison matrix of staging between radiologists reading CT and ground-truth PET and pathological stage. (**G**) Comparison matrix of staging between radiologists reading CT and synthetic PET and pathological stage. (**H**) Consensus matrix of staging between the two radiologists when one radiologist reads true PET and CT compared to another reading synthetic PET and CT. (Reprinted/adapted with permission from Ref. [104].).

A generative adversarial network (GAN)-based CT-to-PET translation framework was developed for patients with lung cancer [104]. Using a 5-point scoring system evaluated by experienced thoracic radiologists (1 = poor, 3 = adequate, 5 = excellent), the average image quality scores were 4.0 for true PET and 3.6 for synthetic PET. For tumor contrast assessment—also rated on a 5-point scale (1 = low, 3 = equal, 5 = high)—the average scores were 4.5 for true PET and 4.1 for synthetic PET. The synthetic PET images showed promising potential for lung cancer diagnosis, staging, and post-treatment prognostication, with performance consistent with that of true PET. Validation of image fidelity and staging accuracy by radiologists is presented in Figure 4 [104].

GANs are widely used for image generation and synthesis. Denoising diffusion probabilistic models (DDPMs) have also demonstrated strong potential for high-quality medical image synthesis. A conditional latent DDPM specifically designed for medical imaging outperformed GANs in terms of image diversity (recall) and achieved comparable or superior image fidelity (precision) [105].

<Summary>

– Generative DL models can synthesize PET images from CT data.

## 5. FAP PET and RLT

### 5.1. Initial Diagnosis and Staging

**Figure 5:**
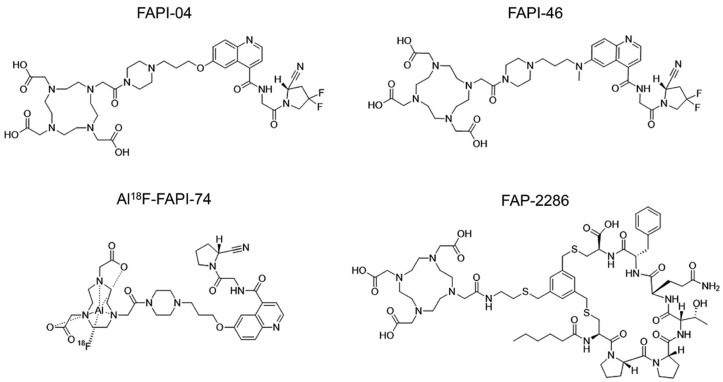
Chemical structures of FAP PET radiopharmaceuticals (Reprinted/adapted with permission from Ref. [106]. Copyright © Society of Nuclear Medicine and Molecular Imaging).

Quinoline-based FAP inhibitors (FAPIs) have been developed to target FAP. More recently, non-quinoline-based FAP-targeted peptide and peptidomimetics have also been introduced. The most commonly used quinoline-based and non-quinoline-based FAP-targeted radiopharmaceuticals are illustrated in Figure 5 [106].

**Figure 6:**
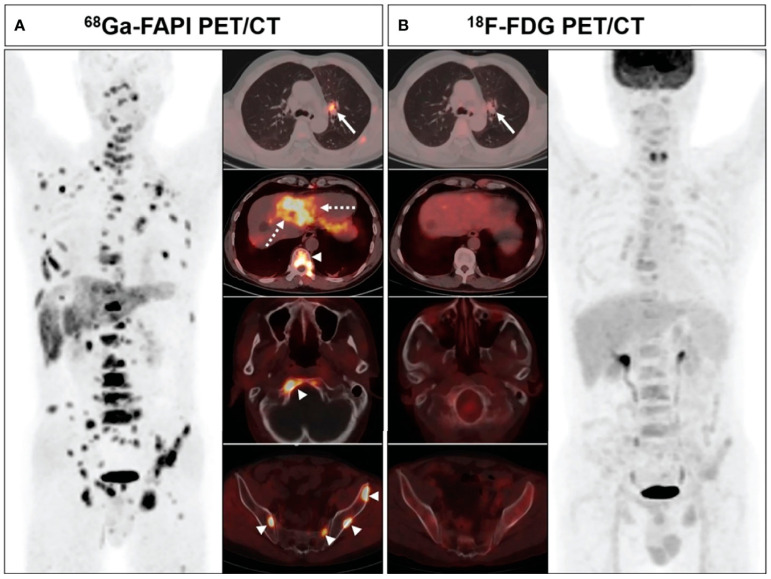
A 63-year-old male diagnosed with lung adenocarcinoma. ^68^Ga-FAPI PET/CT (**A**) showed intense tracer uptake in the primary tumor (solid arrows, SUVmax = 10.0), liver metastasis (dashed arrows, SUVmax = 7.6) and bone metastases (arrows, SUVmax = 8.3–8.5). FDG PET/CT (**B**) showed primary lesion with mild FDG uptake (solid arrows, SUVmax = 3.6), while no significant FDG uptake was showed in liver metastasis and multiple bone metastases (Reprinted/adapted with permission from Ref. [109]. Copyright © 2022 Wu, Deng, Zhong, Wang, Rao, Wang, Chen and Zhang).

Because FAP-targeted radiopharmaceuticals are rapidly cleared via the kidneys and exhibit low uptake in normal tissues, FAP PET provides a higher tumor-to-background ratio (TBR) and reduced physiologic uptake in organs such as the brain and liver, compared to FDG PET [107]. However, proliferating tissues involved in inflammation, regeneration, or degeneration can also express FAP, potentially leading to FP uptake in oncologic FAP PET imaging [108]. Representative ^68^Ga-FAPI PET/CT and FDG PET/CT images illustrating lesion detection in a patient with lung cancer are shown in Figure 6 [109].

A meta-analysis reported Sens of 0.98 (95% CI, 0.88–1.00) for FAP PET/CT and 0.99 (0.74–1.00) for FDG PET/CT in the diagnosis of lung cancer [110]. In a subgroup analysis of NSCLC, the Sens was 0.97 (0.86–1.00) for FAP PET/CT and 0.84 (0.82–0.86) for FDG PET/CT. For detecting nodal and distant metastases in lung cancer, FAP PET/CT demonstrated superior Sens compared to FDG PET/CT (0.99 [0.90–1.00] vs. 0.77 [0.66–0.85]). In the NSCLC subgroup, the Sens were 0.98 (0.88–1.00) for FAP PET/CT and 0.75 (0.62–0.85) for FDG PET/CT. FAP PET/CT also showed higher Sens in detecting metastases in the brain, liver, pleura, and bone, primarily due to its lower physiologic background uptake. Spec could not be evaluated for either modality because of incomplete data regarding true-negative cases.

A prospective study involving 72 patients compared the clinical staging performance of ^68^Ga-DOTA-FAPI-04 PET/CT with that of FDG PET/CT in newly diagnosed NSCLC [111]. ^68^Ga-DOTA-FAPI-04 PET/CT outperformed FDG PET/CT in identifying primary tumors (90% [71/79] vs. 75% [59/79], *p* = 0.01), particularly in non-solid nodular ADC (77.8% [21/27] vs. 40.7% [11/27], *p* = 0.006). For N staging, the SUVmax on ^68^Ga-DOTA-FAPI-04 PET/CT was significantly higher in metastatic lymph nodes than in non-metastatic ones (10.3  ±  5.5 vs. 2.9  ±  1.3, *p* < 0.001), whereas FDG PET/CT showed no significant difference between the two groups (6.2  ±  3.3 vs. 6.1  ±  2.3, *p* = 0.887). Among 98 lymph nodes with pathological confirmation, ^68^Ga-DOTA-FAPI-04 PET/CT demonstrated higher spec (99% vs. 6%), ODA (94% vs. 30%), negative predictive value (NPV) (93% vs. 40%), and PPV (96% vs. 28%), with similar Sens to FDG PET/CT (84% vs. 81%). For M staging, ^68^Ga-DOTA-FAPI-04 PET/CT identified more metastatic lesions than FDG PET/CT (257 vs. 139), particularly in the brain, skull, axial skeleton, lymph nodes, pleura, and liver. However, this did not result in changes to the patients’ overall stage. In 52 patients with complete pathological results, the ODA of ^68^Ga-DOTA-FAPI-04 PET/CT for TNM staging was significantly higher than that of FDG PET/CT (82.7% [43/52] vs. 51.9% [27/52], *p* = 0.001).

Wei et al. compared ^18^F-FAPI PET/CT with FDG PET/CT for diagnosing primary and metastatic lung cancer in a study involving 68 patients [112]. The mean TBR of ^18^F-FAPI PET/CT was significantly lower than that of FDG PET/CT in primary tumors (25.3 ± 14.0 vs. 32.1 ± 21.1, *p* < 0.001). However, the mean TBR of ^18^F-FAPI PET/CT was significantly higher in nodal metastases (7.5 ± 6.6 vs. 5.9 ± 8.6, *p* < 0.001) and bone metastases (8.6 ± 5.4 vs. 4.3 ± 2.3, *p* < 0.001). In the analysis of 548 lesions across 68 patients, ^18^F-FAPI PET/CT demonstrated significantly higher Sens (99% [392/397] vs. 87% [346/397], *p* < 0.001), spec (93% [141/151] vs. 79% [120/151], *p* = 0.004), ODA (97% [533/548] vs. 85% [466/548], *p* < 0.001), and NPV (97% [141/146] vs. 70% [120/171], *p* < 0.001) compared to FDG PET/CT. There was no significant difference in PPV between the two modalities (98% [392/402] vs. 92% [346/377], *p* = 0.57). As a result, the accuracy of TNM staging was significantly higher with ^18^F-FAPI PET/CT than with FDG PET/CT (88% [60/68] vs. 69% [47/68], *p* = 0.004). Incorrect staging occurred in 7 patients for N staging and 1 patient for M staging using ^18^F-FAPI PET/CT, whereas FDG PET/CT led to incorrect staging in 15 patients for N staging and 6 patients for M staging.

In a study involving 73 patients with lung cancer, FAP expression was observed in 97.3% (71/73) of primary tumors, whereas FDG uptake on PET/CT was positive in 87.7% (64/73) (*p* = 0.028) [113]. High FAP expression was detected in 100.0% (15/15) of SqCC, 85.7% (36/42) of ADC, 66.7% (4/6) of large cell neuroendocrine carcinomas, and 40.0% (4/10) of SCLC (*p* < 0.05). Notably, among 12 early-stage ADCs with a ground-glass opacity appearance, 83.3% (10/12) showed FAP expression, while only 25.0% (3/12) were positive on FDG PET/CT.

A recent prospective study compared ^18^F-FAPI-04 PET/CT and FDG PET/CT in 20 patients with clinical stage IA lung ADC [114]. ^18^F-FAPI-04 PET/CT demonstrated significantly higher SUVmax and TBR than FDG PET/CT (3.1 ± 1.6 vs. 1.5 ± 1.0, *p* < 0.001; 4.5 ± 3.8 vs. 2.0 ± 1.8, *p* = 0.04, respectively). The FAP IHC score was positively correlated with the SUVmax of ^18^F-FAPI-04 uptake (r = 0.64, *p* = 0.005).

**Figure 7:**
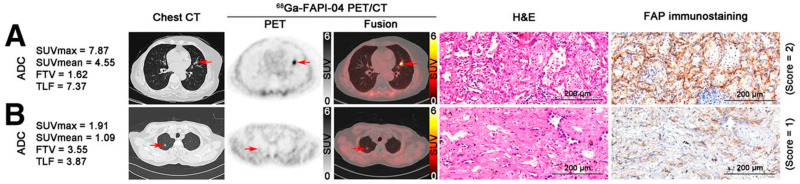
Represent TP (**A**) and FN (**B**) mixed ground glass nodules on chest CT, ^68^Ga-FAPI-04 PET/CT, and corresponding hematoxylin–eosin (H&E) and FAP immunostaining (magnification ×100). (**A**) 76-y-old woman with history of postoperative right-sided breast cancer 1 y previously with ^68^Ga-FAPI–avid ADC (arrows) measuring 18 × 12 × 15 mm in left upper lobe with moderate FAP expression (pT1bN0M0, IA2). (**B**) 76-y-old man with ADC (arrows) measuring 13 × 10 × 20 mm in right upper lobe with visually negative ^68^Ga-FAPI-04 uptake and weak FAP expression (pT1bN0M0, IA2). (Reprinted/adapted with permission from Ref. [115]. Copyright © Society of Nuclear Medicine and Molecular Imaging).

In a study involving 91 patients with NSCLC, the diagnostic Sens and PPV of ^68^Ga-FAPI-04 PET/CT were 96.7% (88/91) and 100% (88/88), respectively [115]. Compared to ADCs, SqCCs demonstrated significantly higher median SUVmax (14.9 vs. 12.8, *p* = 0.031), FAPI-avid tumor volume (26.9 vs. 4.5, *p* < 0.001), and total lesion FAP expression (222.8 vs. 30.1, *p* < 0.001). For nodal metastasis, the Sens, Spec, and accuracy of ^68^Ga-FAPI-04 PET/CT were 72.0% (18/25), 93.1% (108/116), and 89.4% (126/141), respectively. The ODA for TNM staging was significantly higher for ^68^Ga-FAPI-04 PET/CT than for conventional imaging modalities—contrast-enhanced CT, brain MRI, and bone scintigraphy supplemented by CT, MRI, or ultrasound as needed—(82.4% [75/91] vs. 68.1% [62/91], *p* = 0.029). Based on ^68^Ga-FAPI-04 PET/CT findings, the initially planned treatment was modified in 23.1% (21/91) of patients, including 9 cases of downstaging and 12 of upstaging. Representative true-positive (TP) and FN mixed ground-glass nodules on chest CT, ^68^Ga-FAPI-04 PET/CT, and corresponding hematoxylin–eosin and FAP immunostaining are shown in Figure 7 [115].

**Figure 8:**
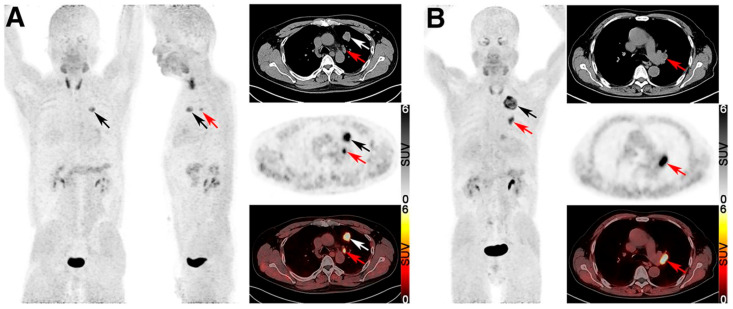
Representative cases of TP and FN lymph nodes on ^68^Ga-FAPI-04 PET/CT. (**A**) PET/CT images showed ^68^Ga-FAPI–avid soft-tissue mass (black and white arrows) and enlarged lymph node (red arrows) in ipsilateral mediastinum (station 5) with intense ^68^Ga-FAPI-04 accumulation (SUVmax, 7.46) in 54-y-old man. Histopathology demonstrated adenocarcinoma with lymph node metastasis (pT2aN2M0, IIIA). (**B**) PET/CT images revealed well-defined solid ^68^Ga-FAPI–avid mass (black arrow) and enlarged lymph nodes in left hilum with intense ^68^Ga-FAPI-04 uptake (SUVmax, 10.33; red arrows) in 62-y-old man. Histopathology confirmed adenocarcinoma with reactive hyperplasia of left hilar lymph node (pT2bN0M0, IIA). (Reprinted/adapted with permission from Ref. [115]. Copyright © Society of Nuclear Medicine and Molecular Imaging).

Representative cases of TP and FP lymph nodes on ^68^Ga-FAPI-04 PET/CT are demonstrated in Figure 8 [115].

<Summary>

– Non-quinoline-based FAP-targeted peptides and peptidomimetics have recently been developed.
– FAP PET offers a high TBR and low physiological uptake in normal organs such as the brain and liver.
– FAP PET demonstrates superior sensitivity to FDG PET in detecting nodal and distant metastases, particularly in NSCLC.
– FAP PET outperforms FDG PET in identifying non-solid nodular lung ADC.

### 5.2. Prediction of PD-L1 Expression and Prognostication

A retrospective study of 75 patients with newly diagnosed lung cancer reported that ^18^F-FAPI PET/CT uptake was positively correlated with PD-L1 expression, whereas FDG PET/CT uptake showed no significant correlation [116]. TBRs were calculated using background uptake in the pulmonary aortic trunk (TBR_blood) and in the contralateral or adjacent non-lesioned lung tissue (TBR_lung). Both TBR_blood and TBR_lung measured on ^18^F-FAPI PET/CT were significantly correlated with PD-L1 expression (r = 0.32, *p* < 0.01; r = 0.26, *p* < 0.05, respectively). Tumors with high PD-L1 expression showed higher ^18^F-FAPI uptake than those with low PD-L1 expression (mean TBR_lung = 36.2 vs. 25.1; mean TBR_blood = 10.8 vs. 8.0). Additionally, TBR_blood on ^18^F-FAPI PET/CT was a significant predictor of PD-L1 expression level, with an AUC of 0.68 (*p* < 0.01).

A study including 59 patients with NSCLC reported that elevated FAP expression was associated with poor tumor differentiation (*p* = 0.06) [117]. Moreover, high FAP expression was significantly associated with worse OS (in percentage: *p* = 0.009; in intensity: *p* = 0.05). A higher percentage of FAP expression was also observed in patients with an elevated peripheral neutrophil-to-lymphocyte ratio (*p* = 0.034), suggesting a link between FAP expression, systemic inflammation, and a suppressed lymphocyte-mediated immune response, which may contribute to tumor progression.

Watanabe et al. compared the prognostic value of ^68^Ga-FAPI-46 PET/CT and FDG PET/CT in staging and restaging of 145 patients with various solid tumor types [118]. A total lesion count of ≥4 on ^68^Ga-FAPI-46 PET/CT was an independent risk factor for poor OS (HR = 1.05, *p* = 0.02). On FDG PET/CT, the presence of hypermetabolic bone metastases independently predicted shorter OS (HR = 3.46, *p* = 0.01). However, no significant prognostic factor was identified in the small subgroup of thoracic cancers, which included 12 lung cancer and 18 pleural mesothelioma patients).

<Summary>

– FAP uptake appears to be positively correlated with PD-L1 expression in lung cancer.
– Elevated FAP uptake may be associated with poorer prognosis in lung cancer.

### 5.3. FAP RLT

Several clinical trials are currently underway to evaluate RLT using FAP-targeted radiopharmaceuticals. Ongoing trials involving patients with solid tumors are summarized in Table 2. NCT04939610 is an ongoing study evaluating ^177^Lu-FAP-2286 in patients with advanced solid tumors, with the estimated completion date for primary outcome data collection set for June 30, 2026. NCT06710756 is a newly initiated phase I/IIa trial investigating α-particle therapy with ^212^Pb-PSV359, guided by ^203^Pb-PSV359 SPECT/CT, in patients with FAP-positive solid tumors.

FAPI has relatively rapid tumor clearance and short tumor retention time [119]. To increase tumor retention, FAP-2286 was developed using cyclic peptides as binding motifs. Its preclinical evaluation for FAP-targeted radionuclide imaging and therapy was conducted by Zboralski et al. [120]. FAP-2286 and its natural nonradioactive metal complexes (^nat^Ga-FAP-2286, ^nat^In-FAP-2286, and ^nat^Lu-FAP-2286) demonstrated potent and selective binding to FAP. Biodistribution studies in mice showed rapid and sustained uptake of ^68^Ga-FAP-2286, ^111^In-FAP-2286, and ^177^Lu-FAP-2286 in FAP-positive tumors, with renal clearance and minimal uptake in normal tissues. Statistically significant antitumor activity was observed, with tumor growth inhibition rates of 111% and 113% (*p* < 0.05) in HEK-FAP tumor-bearing mice treated with 30 or 60 MBq of ^177^Lu-FAP-2286, respectively, compared to the vehicle-treated group, with no significant weight loss. Furthermore, ^177^Lu-FAP-2286 was compared with ^177^Lu-FAPI-46, one of the leading therapeutic FAPI radiotracers. Both agents showed significant antitumor effects, with tumor growth inhibition rates of 108% and 90% (*p* < 0.001), respectively, compared to the vehicle group.

More recently, a prospective study involving nine patients with advanced lung cancer (including four with SqCC, two with ADC, one with adenosquamous carcinoma, one with SCLC, and one with lymphoepithelioma-like carcinoma) reported on the efficacy and safety of ^177^Lu-FAP-2286 therapy following ^68^Ga-FAP-2286 PET/CT imaging [121]. After receiving at least 2 cycles (200 mCi each) of ^177^Lu-FAP-2286 treatment, 4 patients achieved PR, 3 had SD, and 2 experienced PD. The mean Eastern Cooperative Oncology Group performance status score significantly improved from baseline (2.6 ± 0.7 at baseline vs. 1.6 ± 1.2 post-treatment). No grade III/IV hematologic, hepatic, renal, or coagulation toxicities were observed. The most commonly reported adverse effects were loss of appetite and fatigue.

A dual-targeting radiotracer designed to target both FAP expression on CAF and integrin α_v_β_3_ expression on tumor neovasculature has been proposed to enhance the Sens and spec of lung cancer detection [122]. In a pilot exploratory study involving 51 patients with suspected lung cancer, ^68^Ga-FAPI-RGD PET/CT demonstrated a significantly higher detection rate of primary tumors compared to FDG PET/CT (91.4% vs. 77.1%, *p* < 0.05), as well as higher SUVmax (6.9 ± 5.3 vs. 5.3 ± 5.4, *p* < 0.001) and TBR (10.0 ± 8.4 vs. 9.0 ± 9.1, *p* < 0.05). It also showed greater accuracy in assessing mediastinal nodal metastases (99.7% vs. 90.9%, *p* < 0.001) and detected more distant metastatic lesions (254 vs. 220). Additionally, the SUVmax of primary tumors was significantly higher with ^68^Ga-FAPI-RGD than with ^68^Ga-RGD alone (5.8 ± 4.4 vs. 2.3 ± 1.3, *p* < 0.001) [123].

Ceuppens et al. evaluated the therapeutic potential of a FAP-targeting single-domain antibody (4AH29) labeled with either ^225^Ac or ^131^I in immunocompetent mice bearing a human FAP-expressing lung cancer model [124]. ^131^I-GMIB-4AH29 and ^225^Ac-DOTA-4AH29 specifically accumulated in tumors and organs expressing FAP. ^225^Ac-DOTA-4AH29 demonstrated longer tumor retention than ^131^I-GMIB-4AH29. Both agents delayed tumor growth compared to vehicle treatment, with ^225^Ac-DOTA-4AH29 showing a more pronounced delay in tumor progression and improved survival (median survival: 41.5 vs. 36 days). No acute treatment-related toxicities were observed. Notably, PD-L1 expression was elevated in tumors treated with ^225^Ac-DOTA-4AH29 relative to controls. Combining ^225^Ac-DOTA-4AH29 with PD-L1 immune checkpoint blockade further enhanced therapeutic efficacy, resulting in greater tumor growth inhibition and prolonged survival compared to ^225^Ac-DOTA-4AH29 monotherapy or control treatments. In contrast, this synergistic effect was not observed with ^131^I-GMIB-4AH29.

<Summary>

– Ongoing clinical trials are evaluating FAP RLT in FAP-positive solid tumors.
– FAP RLT combined with immune checkpoint inhibitors may enhance therapeutic efficacy.

## 6. Limitations and Future Directions

### 6.1. FDG PET/CT with Radiomics and AI

FDG PET/CT is a standard imaging for the diagnosis, staging, and treatment monitoring of lung cancer. Recent advancements in radiomics and AI—especially ML and DL—have the potential to enhance its utility through automated feature extraction, improved lesion characterization, prognostication, and treatment response prediction. However, despite these promising developments, several limitations still hinder clinical translation.

One of the most significant challenges to the clinical adoption of radiomics and AI in FDG PET/CT is the lack of standardized imaging and feature extraction methods. Variability in image acquisition, reconstruction algorithms, segmentation techniques, and preprocessing steps leads to inconsistent RFs, limiting reproducibility across institutions. Second, many ML/DL models are trained on relatively small, homogeneous datasets, often from single centers. Consequently, these models may not generalize well to external cohorts using different scanners, diverse patient populations, or varied clinical workflows. The absence of external validation and prospective trials further undermines confidence in AI-driven tools. Third, DL models often lack interpretability, making it difficult for clinicians to understand the rationale behind predictions. This “black-box” nature challenges clinical trust, regulatory approval, and routine integration. Fourth, high-quality labeled datasets are essential for training accurate AI models. However, annotating PET/CT data is labor-intensive, and establishing ground truth can be inconsistent or biased, particularly in retrospective studies. Lastly, even well-performing models require substantial effort for clinical integration, including software compatibility, user training, and, notably, regulatory approvals.

Integrating FDG PET/CT-based AI models with clinical, histopathological, and genomic data could enhance predictive performance and enable more personalized lung cancer management. International efforts should prioritize harmonizing RF definitions and image preprocessing protocols to improve reproducibility and support multicenter validation. The development and open sharing of large, annotated PET/CT datasets—including diverse imaging protocols and comprehensive clinical information from multiple institutions and countries—are crucial for training robust AI models. Incorporating explainability into AI algorithms will enhance clinician trust, increase transparency, and facilitate regulatory approval. Finally, prospectively designed, automated AI-driven FDG PET/CT systems are essential for widespread clinical adoption.

### 6.2. FAP PET

FAP PET imaging has been proposed as a promising alternative to FDG PET in various epithelial cancers, particularly in tumors with low glucose metabolism or abundant stromal components, such as certain lung cancer subtypes. Compared to FDG PET, FAP PET offers several advantages, including low background uptake in normal tissues, faster imaging protocols, and potentially improved TBR. However, its clinical utility in lung cancer remains to be fully established.

Although FAP is overexpressed in CAFs within the TME, its expression can be heterogeneous across tumor subtypes and among individual patients. Moreover, FAP expression is not limited to malignancy—it may also be upregulated in non-malignant conditions such as fibrosis, inflammation, and wound healing, potentially leading to FP findings on FAP PET imaging. These factors complicate both patient selection and the interpretation of imaging results.

Most studies on FAP PET have been small, retrospective, or conducted in mixed cancer populations. High-quality, lung cancer–specific prospective data on its role in diagnosis, staging, treatment response assessment, and clinical decision-making remain limited. Furthermore, direct comparisons between FAP PET and FDG PET across different lung cancer subtypes are still underexplored.

A deeper understanding of FAP biology within the lung TME is essential to differentiate malignant from non-malignant FAP expression. Integrating imaging data with transcriptomic or proteomic profiling may aid in identifying biomarkers for improved patient selection and prediction of therapeutic response. Well-designed prospective studies are needed to assess the diagnostic, prognostic, and predictive value of FAP PET in various clinical settings—particularly in FDG-negative tumors, early-stage disease, and treatment monitoring. Direct comparisons with FDG PET, enhanced by AI-driven analysis, will be critical in defining optimal imaging strategies.

### 6.3. FAP RLT

FAP-targeted RLT is an emerging and potentially transformative treatment approach for patients with solid tumors exhibiting high FAP expression. Unlike conventional RT, FAP RLT may provide a therapeutic option for patients with extensive metastatic disease. Moreover, it has the potential for synergistic effects when combined with systemic chemotherapy or immunotherapy. However, this strategy remains in the early stages of clinical development, and several challenges still need to be addressed.

One major limitation affecting the therapeutic efficacy of FAP RLT is the variability of FAP expression across tumor types and among individual patients. Optimal criteria for selecting candidates remain undefined. Currently, patient selection relies primarily on FAP uptake observed on PET imaging, with no established thresholds or validated biomarkers to guide eligibility. Another limitation is that many available FAP-targeted ligands exhibit rapid tumor washout, which restricts their suitability for therapeutic applications using β- or α-emitting radionuclides. This short residence time may also complicate accurate dosimetry planning. Moreover, FAP RLT remains in the early phases of clinical development, with only a limited number of patients treated and scarce long-term outcome data. Potential toxicities to FAP-expressing normal tissues, such as bone marrow and healing tissues, are not yet fully understood. In addition, dosing strategies and the optimal choice of radionuclides have yet to be standardized. Regulatory challenges related to radioligand production and delivery also present significant barriers to widespread clinical implementation.

The development of FAP-targeted ligands with improved tumor retention and optimized pharmacokinetics is essential for effective RLT. Incorporating novel chelators and radionuclides with favorable half-lives and energy profiles may enhance therapeutic efficacy while minimizing toxicity. Quantitative FAP PET parameters could aid in tailoring dosimetry, predicting treatment outcomes, and monitoring response, thereby enabling individualized RLT. FAP RLT should also be investigated in combination with systemic therapies such as immunotherapy, targeted agents, or chemotherapy. Given the immunomodulatory role of CAFs, FAP RLT may synergize with immune checkpoint inhibitors by remodeling the tumor microenvironment. Clinical trials evaluating such combinations could pave the way for novel, personalized treatment strategies. Long-term studies are needed to determine the safety, efficacy, and survival benefits of FAP RLT in patients with lung cancer.

## 7. Conclusions

FDG PET/CT has become an essential tool in the management of lung cancer, serving as a standard imaging modality for initial diagnosis, staging, treatment response assessment, and follow-up evaluation. Recent advancements in radiomics and AI have further enhanced the utility of FDG PET/CT in this setting. However, limitations of FDG—particularly its low spec for malignancy—have driven the development of novel alternative radiotracers.

FAP-targeted radiopharmaceuticals are emerging as a promising alternative to FDG, offering greater spec for tumor-associated stromal components across various cancer types, including lung cancer. They also enable improved PET imaging in tumors with low FDG avidity. When guided by FAP PET as a companion diagnostic, FAP RLT represents a true theranostic approach. As AI-driven PET imaging technologies and therapeutic radiopharmaceuticals are increasingly integrated into clinical practice, they are expected to enhance the precision of diagnosis and therapy in lung cancer, particularly in the era of personalized medicine.

## Figures and Tables

**Table 1:** Recommended time intervals for treatment response assessment using FDG PET/CT [55,58,64,65].

Treatment	Time Interval
Chemotherapy	1–4 weeks
Surgery	6 weeks
Radiofrequency ablation	6–8 weeks
Radiation therapy	12 weeks
Immunotherapy	8–9 weeks

**Table 2:** Current clinical trials involving FAP RLT in solid tumors (recruiting and not yet recruiting).

ID	Official Title	Summary	Start Date
NCT04939610	LuMIERE: A Phase 1/2, Multicenter, Open-label, Non-randomized Study to Investigate Safety and Tolerability, Pharmacokinetics, Dosimetry, and Preliminary Activity of ^177^Lu-FAP-2286 in Patients With an Advanced Solid Tumor	Phase 1 of this study is designed to evaluate the safety and establish the recommended intravenous Phase 2 dose for ^177^Lu FAP 2286 monotherapy. Phase 2 is designed to evaluate the safety and efficacy of ^177^Lu FAP 2286 as monotherapy. Participants in both Phase 1 and 2 will be selected for treatment with ^177^Lu-FAP-2286 based on ^68^Ga-FAP-2286 PET to determine tumor FAP expression (pancreas ductal adenocarcinoma, NSCLC, and breast cancer).	30 July 2021
NCT05723640	Phase I, Open-Label Study of the Safety and Dosimetry of a 4-Dose Regimen of Escalating Doses of ^177^Lu-LNC1004 Injection in Adult Patients With Advanced FAP-Positive Solid Tumors	This study aims to determine the safe and tolerable dose of ^177^Lu-LNC1004 injection. The treatment regimen will consist of a single dose intravenous administration of ^177^Lu-LNC1004 Injection per 6-week cycle, for a total of 2 cycles. The dose per cycle will be fixed for each patient and will be escalated in 4 different dose levels, from 30 mCi to 100 mCi (1.11–3.7 GBq).	3 October 2023
NCT06211647	A Clinical Study to Evaluate the Safety, Tolerability, Dosimetry and Preliminary Efficacy of ^177^Lu-XT117 Injection in FAP-positive Patients With Advanced Solid Tumors	This is a single-center, single-arm clinical study to evaluate the safety, tolerability, dosimetry and preliminary efficacy of ^177^Lu-XT117 injection in patients with FAP-positive advanced solid tumors. Dose escalation will be conducted to determine the dose limiting toxicity, maximum tolerated dose, recommended Phase 2 dose, and to assess dosimetry characteristics.	January 2024
NCT06638034	Diagnosis of Metastatic Tumors on ^68^Ga-FAPI-RGD PET-CT and Radioligand Therapy	This study conducts preliminary clinical transformation and internal irradiation dosimetry research on ^177^Lu-FAPI-RGD—a new dual-targeted ^177^Lu therapeutic drug for the first time in human.	1 May 2024
NCT06636617	Safety, Dosimetry and Treatment Response of ^177^Lu-JH04 in Patients with FAP-Positive Tumors	This is a pilot study to assess the dosimetry, toxicity and response of ^177^Lu-JH04 in patients with FAP-positive tumors. All patients underwent ^68^Ga-FAPI PET/CT for selection and were successively divided into three groups of 3 people each. The three groups received successively an approximately 3.70 GBq (100 mCi), 5.55 GBq (150 mCi) and 7.40 GBq (200 mCi) of ^177^Lu-JH04 up to 4 cycles.	21 August 2024
NCT06710756	A Phase I/IIa Image-Guided, Alpha-Particle Therapy Study of ^203^Pb-PSV359 and ^212^Pb-PSV359 in Patients With Solid Tumors That Are Known to be FAP-Positive	This is a prospective, multi-center open label dose finding, dose expansion study of ^212^Pb-PSV359 in subjects with a positive ^203^Pb-PSV359 SPECT/CT.	28 April 2025
NCT06640413	A Phase I Study to Evaluate the Safety and Preliminary Signs of Efficacy of ^177^Lu-OncoFAP-23 Alone or in Combination With L19-IL2 as a Treatment of Metastatic FAP-positive Solid Tumors	This study is a prospective phase I, open-label, multiple ascending, multi-center dose escalation study to evaluate the safety and preliminary signs of efficacy of ^177^Lu-OncoFAP-23 alone and in combination with the antibody-cytokine conjugate L19-IL2 for the treatment of advanced/metastatic FAP-positive solid tumors.	May 2025 (estimated)
NCT06911489	A Phase 1 Clinical Trial to Evaluate the Safety and Tolerability of ^68^Ga-NRT6020 Injection and ^177^Lu-NRT6020 Injection in FAP-Positive Participants With Advanced Solid Tumors	This study is designed to evaluate the preliminary efficacy, biodistribution, radiation dosimetry, and pharmacokinetics of ^68^Ga/^177^Lu-NRT6020 in FAP-positive patients with advanced solid tumors who have failed or have no available standard therapy.	July 2025 (estimated)

## Abbreviations

The following abbreviations are used in this manuscript:

ADC	adenocarcinoma
AI	artificial intelligence
AJCC	American joint committee on cancer
AUC	area under the receiver operating characteristic curve
CAF	cancer-associated fibroblast
CI	confidence intervals
CMR	complete metabolic response
CNN	convolutional neural network
CR	complete response
DDPM	denoising diffusion probabilistic model
DL	deep learning
DT	decision tree
DTP	dual-time-point
EBUS-FNA	endobronchial ultrasound-guided fine needle aspiration
ES	extensive stage
EGFR	epidermal growth factor receptor gene
FAP	fibroblast activation protein
FAPI	fibroblast activation protein inhibitor
FDG PET/CT	^18^F-fluorodeoxyglucose positron emission tomography/computed tomography
FN	false-negative
FP	false-positive
GAN	generative adversarial network
HRNet	high-resolution representation learning model
IHC	immunohistochemistry
imPERCIST	immunotherapy-modified PERCIST
iRECIST	immune RECIST
imRECIST	immune-modified RECIST
KRAS	Kirsten rat sarcoma
LS	limited stage
MIP	maximum-intensity projection
ML	machine learning
NSCLC	non-small cell lung cancer
ODA	overall diagnostic accuracy
OS	overall survival
PD	progressive disease
PD-1	programmed death 1
PD-L1	programmed death ligand 1
PERCIST	PET response criteria in solid tumors
PFS	progression-free survival
PMD	progressive metabolic disease
PMR	partial metabolic response
PPV	positive predictive value
PR	partial response
RECIST	response evaluation criteria in solid tumors
ResNet	residual network
RF	radiomic feature
RFS	recurrence-free survival
RLT	radioligand therapy
RT	radiation therapy
SBRT	stereotactic body radiation therapy
SCLC	small cell lung cancer
SD	stable disease
Sens	sensitivity
SMD	stable metabolic disease
Spec	specificity
SqCC	squamous cell carcinoma
STP	single-time-point
SUL	SUV corrected for lean body mass
SUV	standardized uptake value
SVM	support vector machine
TBR	tumor-to-background ratio
TP	true-positive
TP53	tumor protein p53
TME	tumor microenvironment
TNM	tumor, node, and metastasis
VOI	volume of interest
